# Personality and Mind-Wandering Self-Perception: The Role of Meta-Awareness

**DOI:** 10.3389/fpsyg.2021.581129

**Published:** 2021-04-15

**Authors:** Miguel Ibaceta, Hector P. Madrid

**Affiliations:** ^1^Alliance Manchester Business School, University of Manchester, Manchester, United Kingdom; ^2^School of Business, Universidad Adolfo Ibáñez, Santiago, Chile

**Keywords:** mind-wandering, personality, meta-awareness, structural equation modeling, daydreaming

## Abstract

Mind-wandering is a psychological process involving the emergence of spontaneous thoughts in daily life. Research has shown that mind-wandering influences diverse psychological outcomes; however, less is known about possible individual differences that may drive mind-wandering. In this study, we argue that personality traits, expressed in neuroticism and openness to experience, may lead to the individual’s self-perception of their mind-wandering activity, due to meta-awareness processes. In a three-wave survey study with 273 college students, we gathered data which supported a positive association of both neuroticism and openness to experience with mind-wandering self-perception, mediated by the individual’s meta-awareness. Thus, this study contributes to the literature on spontaneous thinking by showing that mind-wandering processes may be a function of individual differences expressed in personality traits.

## Introduction

Mind-wandering refers to the shift from events in the external environment to internal, self-generated thoughts, implying that attention is focused on the inner thoughts and feelings (Smallwood and Schooler, 2006). In the last two decades, this cognitive process has been extensively studied, such that the existing research shows that mind-wandering is highly prevalent in our daily life (Killingsworth and Gilbert, 2010; Kane et al., 2017), having influences on different psychological outcomes. For example, getting lost in our thoughts could worsen performance in tasks requiring concentration, whereas the same process can increase creativity (Smallwood and Schooler, 2015). However, other psychological processes associated with spontaneous thinking have been less examined, such as the case of mind-wandering self-perception, namely, the extent to which individuals are aware of their mind-wandering episodes (Schooler, 2002; Chin and Schooler, 2009; Seli et al., 2017). For example, think about when, while reading an entire page of a book, suddenly, you discover you were lost in your thoughts and not paying attention to the printed words you have been scanning. This mind-wandering self-perception is scientifically interesting and should also have practical implications. For example, if the individual is conscious of his/her wandering moments, s/he might use the awareness of mind-wandering to improve performance or mitigate its possible downsides, for instance, increasing concentration on constructing the meaning of the text in the reading example. Thus, a relevant question is what psychological factors lead to mind-wandering self-perception.

In this study, we aim to examine the predictive relationship between personality and mind-wandering self-perception. In general, personality traits have been barely examined as predictors of mind-wandering processes (Robison et al., 2020; Rummel et al., 2020), in favor of other antecedents such as working memory, cognitive capacity, and mindfulness processes (Rummel and Boywitt, 2014; Smallwood and Schooler, 2015). This dearth of knowledge is an important omission, because personality dispositions substantially influence self-regulation of our attention and the content of our thoughts, together with how we process the information gathered from the tasks we perform. Thus, drawing on the Big-5 model of personality, we argue that the traits of neuroticism and openness to experience suffice to explain self-perception of mind-wandering via processes of meta-awareness about our own cognitive process. In the following sections, we elaborate on the theoretical background we employed and present and discuss the study conducted to test our hypotheses.

### Theoretical Background

Personality refers to stable individual differences that describe people’s psychological tendencies (Costa and McCrae, 1992). In the domain of spontaneous thinking, studies have argued that neuroticism and openness to experience are traits that may be related to mind-wandering (Zhiyan and Singer, 1997). Neuroticism involves emotional instability, limited coping with adverse situations, and cognitive rumination tendencies; thus, individuals scoring high on this trait might be prone to wonder about failures and negatively colored experiences (Zhiyan and Singer, 1997; Robison et al., 2017). Neuroticism also makes individuals disposed to strong reflection (e.g., rumination) and decreases executive attention, which is are key conditions to allow the emergence of intrusive cognition, such as the case of mind-wandering thoughts (Robison et al., 2020). In turn, openness to experience is a trait conveying tendencies to develop deep introspection, extensive elaboration of information, and intense thinking processes (McCrae and Sutin, 2009). Therefore, openness may increase the likelihood of mind-wandering due to a richer inner life, expressed in extensive processes of thinking together with plentiful availability of ideas and thoughts (Zhiyan and Singer, 1997; Robison et al., 2020).

As with the *occurrence* of mind-wandering, its self-perception might be determined, in part, by personality. In addition to the psychological processes outlined above, meta-awareness is a psychological process that might explain why neuroticism and openness to experience being related to awareness of our wandering mind. Meta-awareness consists of the process by which individuals attend to their ongoing experience via re-representing their current thoughts (Schooler, 2002). This form of meta-cognition is considered part of a self-monitoring control system that works with the purpose of adjusting behavior to current demands (Chin and Schooler, 2009). In other words, meta-awareness occurs when attention turns inwards and is directed upon the mental processes. We argue that neuroticism and openness to experience may drive meta-awareness because these personality traits are dispositions with substantive implications for the inner psychological domain (McCrae and Costa, 1997; Widiger, 2009). They make individuals prone to reflect on their thinking processes and feelings, together with the content of their thoughts and emotions. Neuroticism drives cognition to the inner world in terms of displeasing contents, while openness does so in relation to reflection about imaginative material.

Meta-awareness, in turn, should be conducive to self-perception of mind-wandering. The experience of mind-wandering can occur consciously (tuning out) or unconsciously (zoning out) (Smallwood and Schooler, 2006). The former implies that individuals are aware of mind-wandering episodes, whereas the latter occurs without the individual’s awareness, except when they are prompted to report their mental states (Smallwood and Schooler, 2015). In contrast, self-perception requires awareness by default because it denotes the extent to which individuals realize episodes of spontaneous thinking. Thus, meta-awareness emerges as a prerequisite for perceiving mind-wandering activity, such that they should be positively related; in other words, a greater level of meta-awareness should be conducive to a higher frequency of mind-wandering self-perception.

Taking the above together, we propose two mediational hypotheses. Specifically, neuroticism will be positively related to meta-awareness, which in turn will be positively related to mind-wandering self-perception (Hypothesis 1), while openness to experience will be positively related to meta-awareness, which in turn will be positively related to mind-wandering frequency (Hypothesis 2).

## Methods

### Procedure and Sample

To test our hypothesis, we conducted a three-wave survey study with a weekly timescale. In the first week, the survey asked participants for their demographic information and ratings about their personality traits. Also, participants responded to a cognitive ability test to use this information as a control variable, due to previous research showing that cognitive ability is related to mind-wandering by means of working memory capacity (Kane and McVay, 2012; Mrazek et al., 2012a). In the second week, the second survey collected ratings about meta-cognition; and finally, in the third week, the last survey asked participants about their mind-wandering self-perception. This three-wave strategy was useful to control issues of common method variance that might bias the statistical estimations observed, by introducing a temporal separation between predictor and criterion variables (Podsakoff et al., 2012). Participants were Chilean college students invited to participate in the study by the first author, who had no previous relationship with them, in exchange for course credits agreed with their lecturer. After their volunteering for the study, participants signed an informed consent form in which the study’s goals and procedures were described, together with stating that participation was voluntary, allowing them to leave the study whenever they decided to, with no negative consequences for them. To ensure voluntary participation, the participants’ lecturer was not present during the application of the surveys. Two hundred and seventy-three students took part in the study. Forty-four percent of participants were female and their average age was 19.01 years (SD = 1.09).

### Measures and Analytical Strategy

In the first week, personality traits were measured using 20 items from the Spanish version of the scales of Benet-Martínez and John (1998), based on the five-factor model of personality, using the stem “I see myself as someone who…”, example items, “worries a lot” (se preocupa mucho por las cosas), “is original, comes up with new ideas” (es original, se le ocurren nuevas ideas) (1: strongly disagree – 5: strongly agree; conscientiousness α = 0.80, extraversion α = 0.74, neuroticism α = 0.76, agreeableness α = 0.61, and openness to experience α = 0.78). Ratings of the five factors were collected to account for all the tendencies that describe the individual’s personality. Cognitive ability was measured with the Spanish version of the Wonderlic Test (Wonderlic, 1992). In the second week, meta-awareness was measured with three items from the cognitive self-consciousness sub-scale from the Metacognitions Questionnaire (Wells and Cartwright-Hatton, 2004), which were translated into Spanish following the procedures described by Brislin (1980), example item, “I constantly examine my thoughts” (constantemente examino mis pensamientos) (1: strongly disagree – 5: strongly agree; α = 0.87). Finally, in the last week, mind-wandering was measured using three items from the Daydreaming Frequency Scale (Giambra, 1993), adapted to capture self-perception of mind-wandering frequency (Preiss et al., 2016), which asks participants about the extent to which their minds wander in their daily life, example item, “I mind wander at work/school” (Mi mente divaga cuando estoy en el trabajo/universidad) (1: never – 5: always, α = 0.75). To determine the validity of this adaptation, we also included a measure of task-unrelated thought using the Mind-Wandering Questionnaire MWQ (Mrazek et al., 2013) (α = 0.82). Thus, we expected that the Daydreaming Frequency Scale would be positively related to MWQ, since these both measures should capture similar constructs. Cronbach’s alpha coefficients are those observed in the data of the study and the translated measures utilized in the study are available in Appendix A.

The data collected with these measures were analyzed with confirmatory factor analysis, in which all the variables involved in the hypothesis testing were loaded in a single model, and correlations among factors were allowed to account for their possible covariance (oblique method) (Brown, 2006). Hypotheses were tested using structural equation modeling with observed variables (path-analysis) (Kline, 2011).

## Results

**FIGURE 1:**
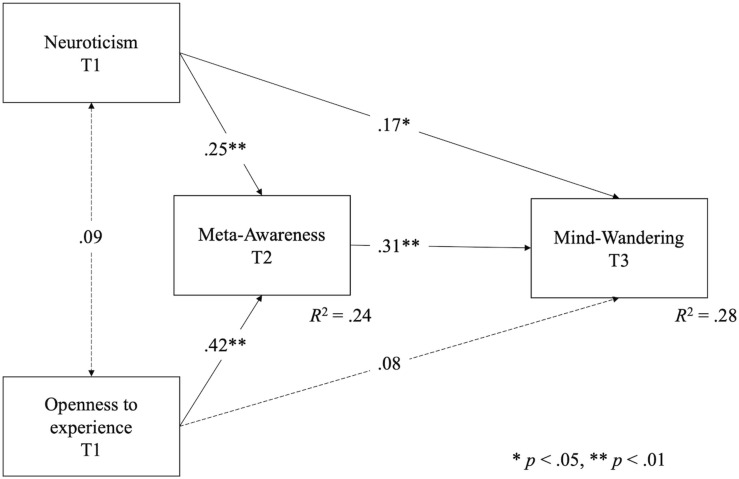
Structural equation modeling for personality, meta-awareness and mind-wandering self-perception.

Results of confirmatory factor analysis for the model described by personality variables, meta-awareness, mind-wandering questionnaire, and mind-wandering self-perception showed acceptable goodness-of-fit, χ^2^ = 587.272(375), *p* < 0.000, RMSEA = 0.05, CFI = 0.91, SRMR = 0.07, which supported the measurement model of the study. Also, measures of the Daydreaming Frequency Scale and the MWQ questionnaire were positively correlated (*r* = 0.60, *p* < 0.01), which supported the convergent validity of our measure of mind-wandering self-perception. Means, standard deviations, correlations, and reliabilities are presented in Table 1. Results of structural equation modeling (Table 2 and Figure 1) showed a positive relationship between neuroticism and meta-awareness, *b* = 0.23, SE = 0.08, *p* < 0.01, which in turn was positively related to mind-wandering self-perception, *b* = 0.27, SE = 0.07, *p* < 0.05, revealing an indirect effect of neuroticism on mind-wandering self-perception by meta-awareness, *b* = 0.06, CI95% [0.01,0.12], *p* = *0.019*. Also, openness to experience was positively related to meta-awareness, *b* = 0.42, SE = 0.09, *p* < 0.01, which in turn, as shown above, was positively related to mind-wandering self-perception, such that openness exerted an indirect effect on mind-wandering self-perception via meta-awareness, *b* = 0.12, CI95% [0.04,0.19], *p* = *0.004*. Therefore, Hypotheses 1 and 2 were supported.

**TABLE 1:** Descriptive statistics, reliabilities, and correlations.

Variable	*M*	SD	1	2	3	4	5	6	7	8	9	10	11
1. Gender (0 = Female, 1 = Male)	0.56	0.50	–										
2. Age	19.01	1.09	–0.00	–									
3. Cognitive ability	112.71	8.63	0.13*	–0.11	–								
4. Extraversion	3.67	0.69	–0.00	–0.00	0.01	(0.74)							
5. Agreeableness	3.97	0.58	–0.06	–0.04	–0.02	–0.03	(0.61)						
6. Conscientiousness	3.84	0.77	−0.22**	–0.04	–0.01	0.07	0.13*	(0.80)					
7. Neuroticism	2.94	0.87	–0.09	0.07	–0.01	–0.09	–0.12	0.09	(0.76)				
8. Openness to experience	3.75	0.84	0.13*	0.05	0.06	0.02	0.14*	–0.01	0.12	(0.78)			
9. Meta-awareness	3.68	0.86	0.10	–0.02	0.02	0.06	–0.07	0.04	0.27**	0.37**	(0.87)		
10. Mind-wandering self-perception	3.32	0.75	–0.02	−0.14*	–0.07	–0.10	0.06	−0.18**	0.25**	0.23**	0.29**	(0.75)	
11. Mind-wandering Questionnaire	3.33	0.73	–0.12	–0.04	–0.11	–0.01	0.08	−0.29**	0.19**	0.08	0.07	0.60**	(0.82)

*N* = 202–273. Reliabilities are displayed along the diagonal in parenthesis. *p < 0.05. **p < 0.01.

**TABLE 2:** Structural equation modeling for personality, meta-awareness and mind-wandering self-perception.

Variable	Meta-awareness T2	Mind-wandering self-perception T3
*Intercept*	4.90 (1.45)	2.65 (2.02)**
***Control variables***		
Gender (0 = Female, 1 = Male)	0.13 (0.07)	−0.07 (0.07)
Age	0.00 (0.14)	−0.13 (0.08)
Cognitive ability	−0.07 (0.07)	−0.09 (0.07)
Conscientiousness T1	−0.03 (0.07)	−0.17 (0.07)*
Agreeableness T1	−0.07 (0.06)	0.11 (0.07)
Extraversion T1	0.04 (0.07)	0.00 (0.07)
***Main effects***		
Neuroticism T1	0.25 (0.08)**	0.17 (0.07)*
Openness to experience T1	0.42 (0.08)**	0.08 (0.08)
Meta-awareness T2		0.31 (0.08)**
***Indirect effects*** CI95%		
Neuroticism		0.08* [0.02,0.14] *p* = 0.01
Openness to experience		0.13** [0.04,0.22], *p* = 0.00
Effect size (R^2^)	0.24	0.28
χ^2^ = (df)	26.50 (12)	

*N* = 178. Standardized estimates. *p < 0.05, **p < 0.01. Standard errors are displayed in parenthesis and confidence intervals in brackets.

## Discussion

This study has shown that personality traits are related to mind-wandering self-perception by means of meta-awareness. Specifically, neuroticism and openness to experience were positively related to the awareness of one’s own thoughts, which in turn were positively related to self-perception of one’s own mind-wandering activities. According to our theorization, these processes are explained because neuroticism and openness to experience are traits that make individuals prone to focusing on their inner world, in terms of concerns in the case of neuroticism and imagination in that of openness, which is the realm of meta-awareness and mind-wandering. The other individual differences included in the model as covariables, namely, conscientiousness, extraversion, agreeableness and cognitive ability were not related to meta-awareness or mind-wandering self-perception. These results are different from those observed in previous studies in which, for example, evidence shows that the greater the conscientiousness and mental ability, the lesser the mind-wandering (Zhiyan and Singer, 1997; Randall et al., 2014). These differences, however, are likely because of the characteristic of our sample composed of college students, in which there would be a range restriction in the measurement of these individual differences.

This study, therefore, contributes to the research on mind-wandering by identifying individual differences that may be involved in how self-awareness of spontaneous thinking emerges in daily life. Thus far, theory and empirical studies at the individual level of analysis have concentrated on how mental ability and cognitive capacity lead to episodes of mind-wandering, influencing thereby task performance. However, the same research has not paid enough attention to whether personality also acts as a predictor of mind-wandering, especially in terms of its self-perception. Thus, we address these issues, proposing a model in which personality is a possible cause, through meta-cognitive mechanisms, of a wandering mind. The results observed here are consistent with previous studies on daydreaming, a form of mind-wandering loaded with imaginative contents, in which personality was also observed as one of its predictors (Zhiyan and Singer, 1997; Robison et al., 2020).

This study has limitations which need to be discussed. We assume a causal relationship between personality traits and self-perception of mind-wandering via meta-awareness, but this is only possible in theoretical terms because our data relies on a survey design. Also, our source of information was based on self-reports of participants for all the variables measured, which might introduce bias in the results due to common method variance issues (Podsakoff et al., 2012). We controlled for this limitation by separating measurement of the variables over 3 weeks; however, issues of method variance might still be present in the model presented. Another related limitation implies that retrospective self-reports for measuring mind-wandering self-perception might be biased, because the information captured with this methodology might account only for mind-wandering events remaining in the memory, but not those forgotten (Bradburn et al., 1987). Moreover, participants in the study were individuals in their early adulthood, which might introduce bias in the results because it is established that mind-wandering has a negative correlation with age (Maillet et al., 2018; Giambra, 2000). Therefore, future research based on experimental and longitudinal designs, using ecological momentary assessment or experience sampling methodology (Shiffman et al., 2008; Bolger and Laurenceau, 2013), with diverse samples of participants, will be informative about how robust the results observed here are.

Finally, there are opportunities for future research to expand the results of this study. Personality traits other than those described by the five-factor model of personality might be explored as antecedents of mind-wandering in addition. This is the case for the traits of need for cognition and need for cognitive closure, which describe the extent to which individuals engage in deeper information processing and introspection (Cacioppo and Petty, 1982). Thus, additional research on these issues will be valuable to gain a broader understanding of mind-wandering processes. Furthermore, we have supported the view that meta-awareness can be conducive to the self-perception of mind-wandering; however, other studies have also argued that meta-awareness would be positively related to mindfulness, i.e., the focus of attention on the present experience, which is the opposite of mind-wandering (Dahl et al., 2015). These conflicting arguments might be solved by taking into account Mrazek et al.’s (2012b) and Hasenkamp et al.’s (2012) proposals suggesting that in the process of mindfulness, mind-wandering awareness comes first, and after that, individuals direct their thoughts to their current experience. The latter stresses that additional longitudinal research will be valuable to disentangle these processes. Finally, we focused on self-perception of mind-wandering activity in general, but studies on whether personality is related to specific forms of spontaneous thoughts would also be informative. This is the case for daydreaming, time mental travel, and reflection about concerns.

To sum up, in this study we have examined whether personality is a driver of self-perception of mind-wandering, showing that neuroticism and openness to experience should play a role in this. We trust that future research will use this knowledge to expand our understanding of the fascinating process of spontaneous thinking.

## Data Availability Statement

The raw data supporting the conclusions of this article will be made available by the authors, without undue reservation.

## Ethics Statement

Ethical review and approval was not required for the study on human participants in accordance with the local legislation and institutional requirements. The patients/participants provided their written informed consent to participate in this study.

## Author Contributions

MI and HM designed, analyzed, and wrote up the research. MI collected the data. Both authors contributed to the article and approved the submitted version.

## Conflict of Interest

The authors declare that the research was conducted in the absence of any commercial or financial relationships that could be construed as a potential conflict of interest.

## Appendix A

### Personality Traits

I see myself as somenone who… (1: Strongly Disagree Agree – Strongly Agree):

Me veo a mismo como una persona que… (1: En total desacuerdo – 5: En total acuerdo):

• Is outgoing, sociable (Es sociable, le gusta conocer a otras personas)
• Is talkative [Es bien hablador(a)]
• Has an assertive personality (Tiene una personalidad dominante)
• Is dominant, acts as a leader (Es influyente, actúa como un líder)
• Is compassionate, has a soft heart [Es compasivo(a), bondadoso]
• Is helpful and unselfish with others [Es generoso(a) y ayuda a los demás]
• Is respectful, treats others with respect [Es respetuoso(a), cortés con las demás personas]
• Has a forgiving nature (Le es fácil perdonar a otras personas)
• Is systematic, likes to keep things in order (Es organizado(a), le gusta mantener las cosas en orden)
• Is efficient, gets things done (Es eficiente, termina las tareas)
• Is persistent, works until the task is finished (Es persistente, trabaja hasta que la tarea esté finalizada)
• Is reliable, can always be counted on (Es responsable)
• Can be tense [Con frecuencia se pone tenso(a)]
• Worries a lot (Se estresa mucho por las cosas)
• Often feels sad (A menudo se siente triste)
• Is temperamental, gets emotional easily (Es temperamental, de humor cambiante)
• Is curious about many different things (Siente curiosidad por diferentes temas)
• Is complex, a deep thinker (Es un pensador profundo)
• Is fascinated by art, music, or literature (Le gusta mucho el arte, la música, o la literatura)
• Values art and beauty (Valora el arte y la belleza)

### Meta-Awareness

Indicate your agreement with the following statements (1: Strongly Disagree Agree – Strongly Agree):

Indique su grado de acuerdo con las siguientes afirmaciones (1: En total desacuerdo – 5: En total acuerdo):

• I think a lot about my thoughts (Reflexiono mucho acerca de mis pensamientos)
• I constantly examine my thoughts (Constantemente estoy pendiente de mis pensamientos)
• I pay close attention to the way my mind works (Le presto mucha atención a la forma en que pienso)

### Mind-Wandering Self-Perception

Indicate how frequently you carry out the following activities (1: Never – Always):

Indique con qué frecuencia usted realiza las siguientes actividades (1: Nunca – 5: Siempre):

• When I am at a meeting or show that is not very interesting, I mind wander rather than pay attention (Mi mente divaga cuando estoy en una reunión o presentación que no es muy interesante)
• I mind wander at work/school (Mi mente divaga cuando estoy en el trabajo/universidad)
• I lose myself in active mind-wandering (Mi mente se pierde en sus pensamientos)
